# Predictors of shortages of opioid analgesics in the US: Are the characteristics of the drug company the missing puzzle piece?

**DOI:** 10.1371/journal.pone.0249274

**Published:** 2021-03-31

**Authors:** Rosa Rodriguez-Monguio, Mahim Naveed, Enrique Seoane-Vazquez

**Affiliations:** 1 Department of Clinical Pharmacy, University of California San Francisco (UCSF), San Francisco, California, United States of America; 2 Medication Outcomes Center, University of California San Francisco (UCSF), San Francisco, California, United States of America; 3 Philip R. Lee Institute for Health Policy Studies at the University of California San Francisco (UCSF), San Francisco, California, United States of America; 4 Department of Biomedical and Pharmaceutical Sciences, Chapman University School of Pharmacy, Irvine, California, United States of America; 5 Economics Science Institute, Argyros School of Business and Economics, Chapman University, Irvine, California, United States of America; Centers for Disease Control and Prevention, UNITED STATES

## Abstract

**Background:**

Shortages of opioid analgesics are increasingly common, interfere with patient care and increase healthcare cost. This study characterized the incidence of shortages of opioid analgesics in the period 2015–2019 and evaluated potential predictors to forecast the risk of shortages.

**Methods:**

This was an observational retrospective study using the US Food and Drug Administration (FDA) drug shortages data. All FDA approved opioids were included in the study. Opioid analgesics were identified using the FDA National Drug Codes (NDC) and classified according to the Drug Enforcement Administration (DEA) schedule. We conducted Least Absolute Shrinkage and Selection Operator logistic regression analysis to assess direction of the association between risk of shortage and potential predictors. We used multivariable penalized logistic regression analysis to model predictors of shortages. We split the dataset into training and validation sets to evaluate the performance of the model.

**Findings:**

The FDA approved 8,207 unique NDCs for opioid analgesics; 3,017 (36.8%) were in the market as of April 30, 2019 and 91(3.0%) of them were listed as in shortage by the FDA. All NDCs in shortage were schedule II opioids; 86 (94.5%) were injectable and 84 (92.3%) generics. There were 418 companies with at least one opioid NDC listed by the FDA. Three companies accounted for more than 4 in 5 of the schedule II active injectable opioids. For each unit increase in the number of prior instances of shortages of a company, the likelihood of an NDC shortage for that company increased by 3.4%. For each unit increase in number of NDCs marketed by a company, the odds of an NDC shortage for that company decreased by 1%.

**Conclusions:**

In the period 2015–2019, shortages of opioid analgesics disproportionally impacted schedule II and injectable opioids. The risk of shortage of opioid analgesics significantly increased with the incidence of previous instances of shortages of a manufacturing company and decreased with the number of NDCs marketed by a company. The characteristics of the manufacturing company, rather than the number of companies, might be the missing piece to the complex puzzle of drug shortages in the US.

## Introduction

The number of drug shortages has risen steadily in the United States (US) since 2005 [1, 2]. Drug shortages are a significant ongoing threat to patient safety by increasing patient morbidity and mortality [3–5]. Due to drug shortages, clinicians often have to turn to therapeutic alternatives, forms or strengths that may be less effective or with which they may not be familiar, increasing the risk of medication errors [6]. Drug shortages may trigger sudden changes in formularies and clinical decision-support systems that disrupt the continuity of patient care [5]. In addition, drug shortages increase health care costs associated with changes to more expensive therapeutic alternatives and drug formulations, tracking inventories, relocating units left in inventory and stockpiling, and other drug procurement strategies that often follow the reporting of a shortage. Drug shortages often result in wasteful healthcare spending for insurers, providers and patients [4, 7].

Shortages of opioid analgesics have become increasingly common, a trend that is projected to continue [8]. These shortages are related to Drug Enforcement Administration opioid manufacturing quotas, in response to the opioid-overdose epidemic and manufacturing problems, including violations in good manufacturing practices [9]. Shortages of opioid analgesics have the potential to negatively impact the care provided to surgical patients by compromising pain control after surgery and increasing opioid-related adverse events arising from drug substitutions [10]. The shortage of opioid analgesics is not expected to be resolved in the near future. Hence, healthcare settings will need to implement prevention and mitigation strategies. Preventing and mitigating the impact of drug shortages require a thorough understanding of the pharmaceutical sector, the drug market and market dynamics.

Prior studies assessed overall trends of pharmaceutical shortages in the US for specific drug classes including antimicrobials, cancer chemotherapy, and cardiovascular and neurologic drugs [1, 2, 4, 8, 11]. Despite its high prevalence and incidence, little is known about the characteristics of shortages of opioid analgesics. This study characterized and assessed the incidence of shortages of opioid analgesics in the US in the period 2015–2019 and evaluated potential predictors to forecast the risk of shortages.

## Materials and methods

This was an observational retrospective study. Shortage data were collected from the US Food and Drug Administration (FDA) website [12]. We collected FDA data for the first reported shortage date during the period 2015–2019. FDA approved opioids data including abbreviated new drug applications (ANDAs) and new drug applications (NDAs) were derived from the electronic version of the “Approved Drug Products with Therapeutic Equivalence Evaluations” (Orange Book). Opioid analgesics were identified by their national drug codes (NDCs) using the FDA NDC directory and classified according to the Drug Enforcement Administration (DEA) schedule [13]. Active ingredient, strength, dosage form, generic and trade names, market discontinuation indicator, unit-dose indicator, company identifier, and drug price data were derived from the IBM Micromedex Red Book (RB) [14]. Opioid shortages data and opioid analgesics information were linked using the NDC to create a unique dataset.

This study used the NDC as the unit of analysis. The NDC is a unique three-segment number that identifies the manufacturer, the drug product, and package. Study categorical variables included administration route, active ingredient, and formulation (extended release and immediate release opioid), form (tablets, capsules, patches, suppositories), manufacturer name, NDC market discontinuation indicator, unit dose and fixed-dose drug combination indicators, generic and brand name indicator. Drug administration routes included injectables (epidural, intramuscular, intrathecal, intravenous), oral (tablets, pills) and other (nasal, rectal, transdermal). Continuous variables included strength, drug unit and package average wholesale price (AWP), and number of manufacturers, ANDAs, NDAs and NDCs by market status (active and discontinued) and number of manufacturers for any given NDC and for any given number of active ingredients. The company risk of shortage was defined as the number of NDCs a company had in shortage prior to a given NDC being in shortage. We hypothesized that the greater the number of NDCs a company had in shortage, the greater the likelihood that the company will have a future NDC in shortage.

We used Chi-square two-independent sample test to assess significant differences between incidence of shortages and categorical variables. We used Wilcoxon non-parametric tests of medians for non-normally distributed continuous variables. We conducted bivariate logistic regression analysis to assess association between risk of shortage and potential predictors. In addition, we conducted Least Absolute Shrinkage and Selection Operator (LASSO) logistic regression analysis to assess direction of the association between risk of shortage and potential predictors. Last, we used multivariable penalized logistic regression analysis to model predictors of shortages. Overall, 44 potential predictive variables were assessed in the LASSO regression analysis including drug administration route, NDCs marketed and discontinued, extended release and fixed-dose combination drug indicator, generic and unit dose indicator, DEA schedule, number of companies manufacturing any given active ingredient, strength and route, number of NDCs marketed by any given company and drug unit and package AWP price and company risk and combinations of these predictors. Of the potential predictors, coefficients of 24 variables were not penalized to zero and therefore, remained significant predictors. Final regression analysis included 4 variables that were independently statistically significant predictors of shortages of opioids.

**Fig 1:**
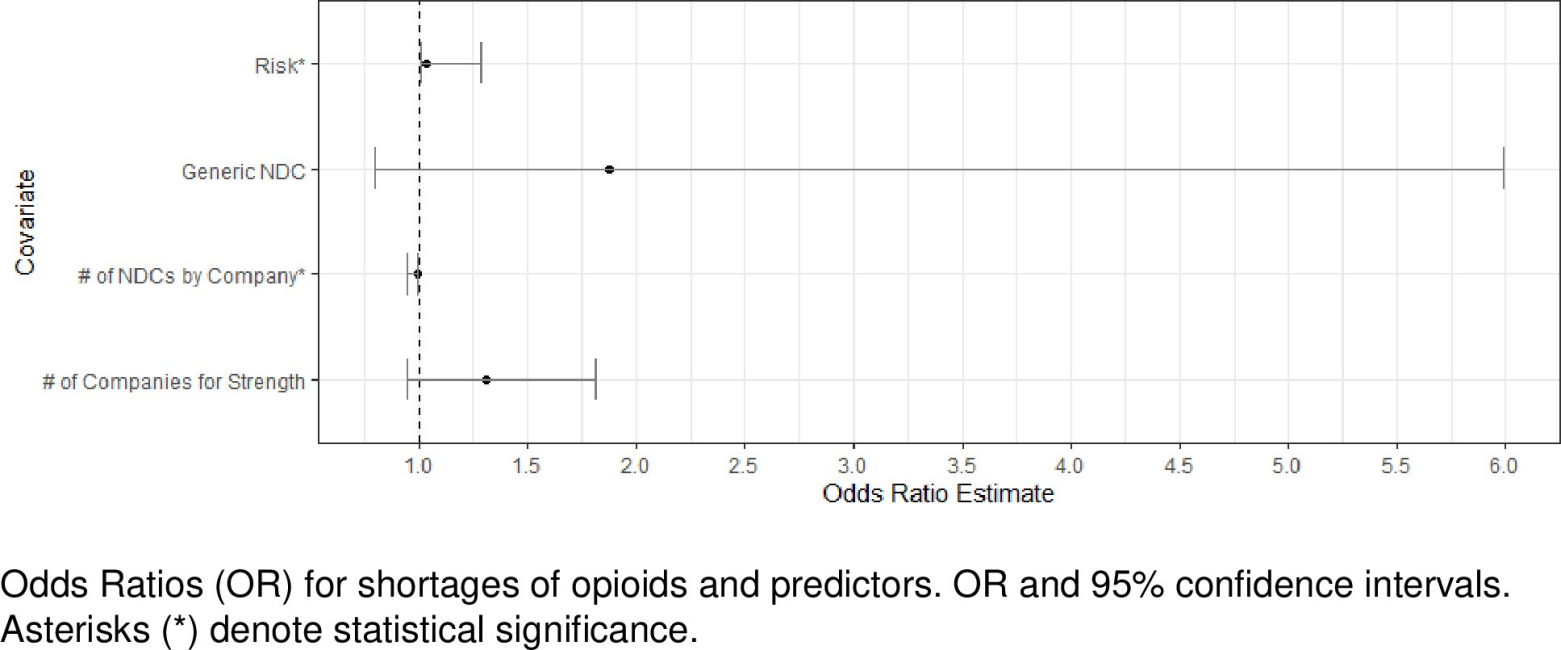
Shortages of opioids: Odds ratios estimates.

**Fig 2:**
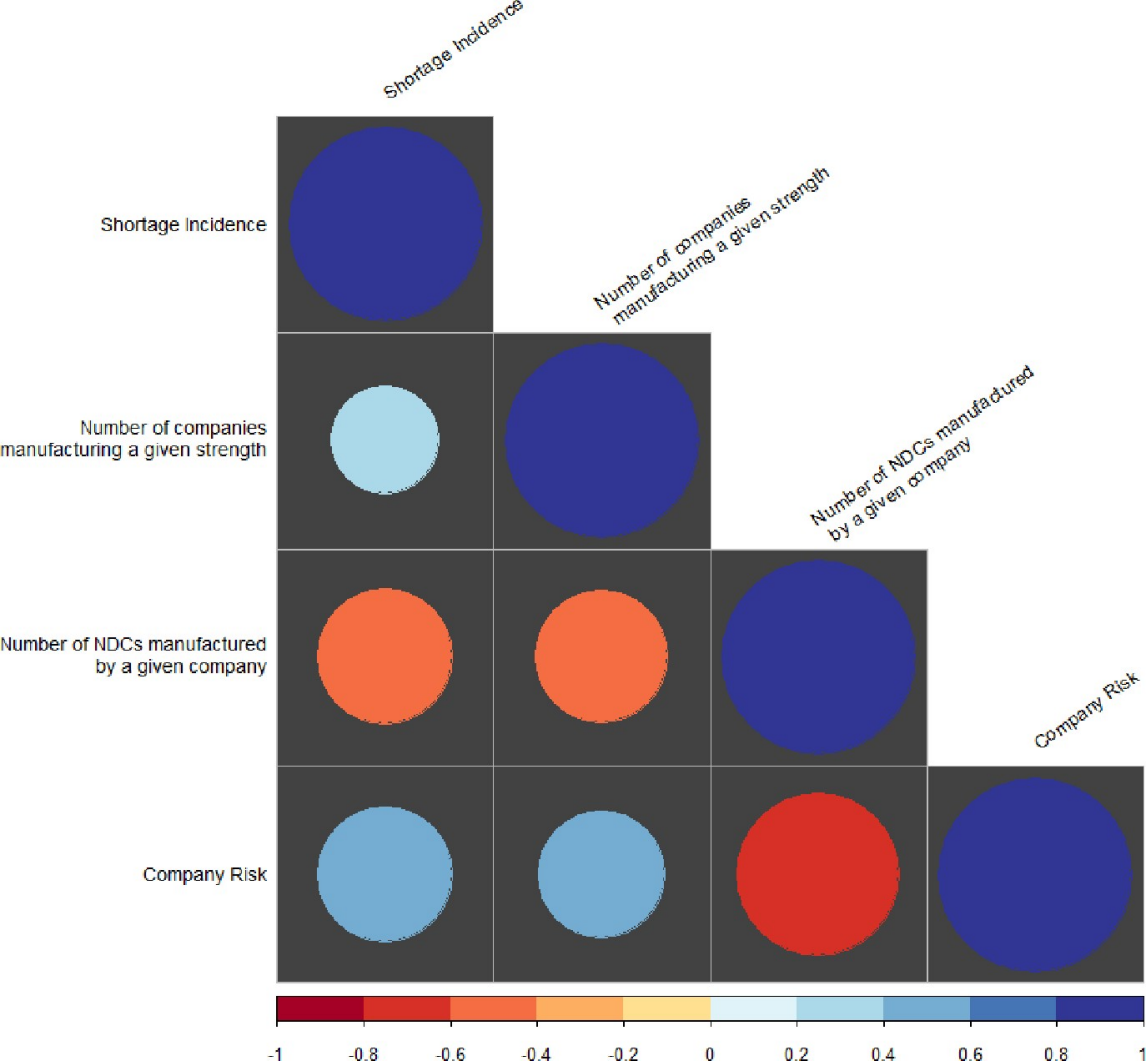
Kendall’s tau correlation coefficients for predictors of shortages.

Our penalized logistic regression model followed a bias-reduced Generalized Linear Model adjustment that uses Firth’s correction to address perfect separation for low incidence observations. We reported log odds (beta estimates), standard error and odds ratios (exponentiated beta estimates) with 95% confidence intervals (CI) for predictors of shortages (Fig 1). We used Kendall’s tau correlation coefficients to assess potential correlation among covariates (Fig 2). In addition, we assessed multicollinearity in the penalized logistic regression model using the variance inflation factor (VIF < 5) and correlation matrix of parameter estimates (±1).

Last, we split the dataset into training and validation sets using 70:30 ratio, respectively, to evaluate the performance of the model. The training data sample was used to fit the model. Model fit was assessed using Hosmer-Lemeshow (H-L) statistic (H-L > 0.05). The validation set was used to assess model’s predictive performance using the area under the receiver operator characteristic (ROC) curve. We also computed confusion matrix using the cut-off threshold probability identified by ROC curve to assess classification accuracy of the model. We used odds ratios and beta estimate coefficient plots to represent penalized logistic regression model results to visualize direction and strength of association between risk of shortages and covariates. Analyses were performed using R software (version 3.6.2, R Foundation). A p-value < .05 was considered statistically significant.

## Results

### Incidence of shortages of FDA approved opioid analgesics

The FDA listed 8,207 unique NDCs for opioid analgesics (Table 1). Overall, 5,726 (69.8%) of the FDA approved opioids were classiffied by the DEA as schedule II. Of the total (105, 1.3%) NDCs in shortage at the FDA as of April 2019, 104 (99.0%) were schedule II, 89 (84.8%) injectable, and 91(86.7%) generics. Overall, 418 companies had at least one opioid NDC listed by the FDA. Of them, 183 (43.7%) had at least one active opioid NDC as of April 30, 2019 and 16 (3.8%) companies were marketing schedule II injectable opioids. The median (IQR) number of NDCs per company was 6.0 (2.0, 18.0) for all NDCs, and 5.0 (2.0, 15.5) for active NDCs. The median number of schedule II injectable NDCs per company was 15.0 (3.5, 36.0); all schedule II injectable NDCs remained in the market as of April 30, 2019. Likewise, the median (IQR) number of companies per strength was 2.0 (1.0–4.0) for all NDCs and 1.0 (1.0–3.0) for active NDCs. The median (IQR) number of companies per schedule II opioid injectable manufacturing a given strength was 1.0 (1.0–1.0) for all NDCs; the strength remained in the market as of April 30, 2019.

**Table 1:** FDA approved opioid NDCs and shortages, 2015–2019.

	NDCs	% NDCs	FDA Shortages	% of NDC	% of FDA Shortages	p value
**NDCs**	8,207		105	1.3%		
**Strengths**	877		37	4.2%		
**Companies**	418		11	2.6%		
**DEA Schedule**						<0.001
** CII**	5,726	69.8%	104	1.8%	99.0%	
** CIII**	762	9.3%				
** CIV**	757	9.2%	1	0.1%	1.0%	
** CV**	962	11.7%				
**Route**						<0.001
** Oral**	4,881	59.5%	16	0.3%	15.2%	
** Injectable**	2,289	27.9%	89	3.9%	84.8%	
** Other**	1,037	12.6%				
**Extended release formulation**	944	11.5%	16	1.7%	15.2%	0.292
**Fixed-dose drug combination**	4,734	57.7%				
**Generic drugs**	6,943	84.6%	91	1.3%	86.7%	0.649
**Unit dose**	597	7.3%				0.007
**Active ingredient**						<0.001
** alfentanil hydrochloride**	25	0.3%				
** buprenorphine**	137	1.7%				
** buprenorphine hydrochloride**	67	0.8%				
** codeine phosphate**	1340	16.3%				
** diphenoxylate hydrochloride**	123	1.5%				
** fentanyl**	275	3.4%				
** fentanyl citrate**	979	11.9%	21	2.1%	20.0%	
** hydrocodone**	4	0.0%				
** hydrocodone bitartrate**	1276	15.5%				
** hydrocodone polistirex**	27	0.3%				
** hydrocodone tannate**	2	0.0%				
** hydromorphone hydrochloride**	604	7.4%	31	5.1%	29.5%	
** meperidine hydrochloride**	260	3.2%	1	0.4%	1.0%	
** methadone hydrochloride**	118	1.4%	2	1.7%	1.9%	
** morphine sulfate**	1131	13.8%	43	3.8%	41.0%	
** morphine sulfate liposome**	7	0.1%				
** opium**	24	0.3%				
** oxycodone**	5	0.1%				
** oxycodone hydrochloride; oxycodone terephthalate**	35	0.4%				
** oxycodone hydrochloride**	596	7.3%				
** pentazocine lactate**	13	0.2%	1	7.7%	1.0%	
** remifentanil hydrochloride**	19	0.2%	6	31.6%	5.7%	
** sufentanil citrate**	93	1.1%				
** tramadol hydrochloride**	248	3.0%				
** All other**	799	9.7%				
**Company (Top 10)**						<0.001
** Teva Pharmaceuticals USA**	351	4.3%	3	0.9%	2.9%	
** Hospira, Inc.**	231	2.8%	37	16.0%	35.2%	
** Specgx, LLC**	154	1.9%				
** Par Pharmaceutical**	146	1.8%	5	3.4%	4.8%	
** Apotex Corp.**	150	1.8%				
** Amneal Pharmaceuticals Inc.**	124	1.5%				
** Mylan Pharmaceuticals Inc.**	98	1.2%	9	9.2%	8.6%	
** Hikma Pharmaceuticals Inc.**	92	1.1%	12	13.0%	11.4%	
** Fresenius Kabi USA, LLC**	55	0.7%	23	41.8%	21.9%	
** Akorn, Inc.**	60	0.7%	9	15.0%	8.6%	
** All other companies**	6,365	77.6%				
**AWP unit price median(IQR)**	0.6 (0.2, 3.3)		2.9 (1.2, 4.3)			<0.001
**AWP package price median (IQR)**	62.5 (22.9, 239.7)		82.7 (30.7, 170.0)			0.05
** Missing**	481	5.9%				
**Companies per strength (median(IQR)**	2.0 (1.0, 4.0)		8.0 (3.0, 13.0)			<0.001
**NDCs per company (median(IQR))**	6.0 (2.0, 18.0)		42.0 (11.0, 54.5)			0.005

Injectable included injection, epidural, intramuscular, intrathecal, intravenous. Other administration routes included buccal mucosa, nasal, oromuscular, rectal, subcutaneous, transdermal. Other active ingredient included benzohydrocodone, butarphanol tartrate, codeine, dezocine, difenoxin hydrochloride, dihydrocodeine bitartrate, levomethadyl acetate hydrochloride, levorphanol tartrate, nalbuphine hydrochloride, terephthalate, oxymorphone hydrochloride, paregoric, pentazocine hydrochloride, propoxyphene hydrochloride, propoxyphene napsylate, tapentadol hydrochloride.

### Shortages of opioids in the US market

Out of 8,207 unique NDC opioids, 3,017 (36.8%) were active as of April 30, 2019 (Table 2). In the study period, 91(3.0%) of the active NDCs were listed by the FDA as in shortage. All active NDCs in shortage were schedule II opioids; 86 (94.5%) were injectable and 84 (92.3%) generic opioids. There were no statistically significant differences in the characteristics of generic opioids in shortage and not in shortage. There were not shortages of fixed-dose combinations or unit dose opioids. There were significant differences in the incidence of shortages by active ingredient. The incidence of shortages was higher for morphine (32, 35.2%), hydromorphone hydrochloride (31, 34.1%), and fentanyl citrate (19, 20.9%). Likewise, the incidence of shortages also varied by company. Overall, 91(3.0%) of the active opioid NDCs in shortage were registered by 7 (3.8%) out of 183 companies with active NDC opioids.

**Table 2:** Opioid NDCs marketed and shortages, 2015–2019.

	NDCs	% NDC Marketed	% NDCs	p value	FDA Shortages	% NDC	% FDA Shortages	p value
**NDCs**	3,017		36.8%		91	3.0%		<0.001
**Strengths**	524		59.7%		30	5.7%		
**Companies**	183		43.8%		9	4.9%		
**DEA Schedule**				<0.001				<0.001
** CII**	2,519	83.5%	44.0%		91	3.6%	100.0%	
** CIII**	234	7.8%	30.7%					
** CIV**	171	5.7%	22.6%					
** CV**	93	3.1%	9.7%					
**Route**				<0.001				<0.001
** Oral**	1,301	43.1%	26.7%		5	0.4%	5.5%	
** Injectable**	1,030	34.1%	45.0%		86	8.3%	94.5%	
** Other**	686	22.7%	66.2%					
**Extended release formulation**	495	16.4%	52.4%	<0.001	5	1.0%	5.5%	0.007
**Fixed-dose drug combination**	1,155	38.3%	31.8%	<0.001				
**Generic drugs**	2,690	89.2%	38.7%	<0.001	84	3.1%	92.3%	0.418
**Unit dose**	270	8.9%	45.2%	<0.001				0.004
**Active ingredient**				<0.001				<0.001
** alfentanil hydrochloride**	11	0.4%	44.0%					
** buprenorphine**	121	4.0%	88.3%					
** buprenorphine hydrochloride**	48	1.6%	71.6%					
** codeine phosphate**	144	4.8%	10.7%					
** diphenoxylate hydrochloride**	20	0.7%	16.3%					
** fentanyl**	196	6.5%	71.3%					
** fentanyl citrate**	563	18.7%	57.5%		19	3.4%	20.9%	
** hydrocodone bitartrate**	417	13.8%	32.7%					
** hydrocodone polistirex**	12	0.4%	44.4%					
** hydromorphone hydrochloride**	314	10.4%	52.0%		31	9.9%	34.1%	
** meperidine hydrochloride**	73	2.4%	28.1%		1	1.4%	1.1%	
** methadone hydrochloride**	77	2.6%	65.3%		2	2.6%	2.2%	
** morphine sulfate**	390	12.9%	34.5%		32	8.2%	35.2%	
** opium**	7	0.2%	29.2%					
** oxycodone**	5	0.2%	100.0%					
** oxycodone hydrochloride**	283	9.4%	47.5%					
** remifentanil hydrochloride**	15	0.5%	78.9%		6	40.0%	6.6%	
** sufentanil citrate**	42	1.4%	45.2%					
** tramadol hydrochloride**	144	4.8%	58.1%					
** Other**	135	4.5%	16.9%					
**Company (Top 10)**				<0.001				<0.001
** Teva Pharmaceuticals USA**	113	3.7%	32.2%		3	3.3%	2.7%	
** Hospira Inc**	125	4.1%	54.1%		35	38.5%	28.0%	
** Specgx LLC**	143	4.7%	92.9%					
** Par Pharmaceutical**	58	1.9%	39.7%					
** Apotex Corp**	93	3.1%	62.0%					
** Amneal Pharmaceuticals Inc**	76	2.5%	61.3%					
** Mylan Pharmaceuticals Inc**	65	2.2%	66.3%		9	9.9%	13.8%	
** Hikma Pharmaceuticals USA Inc**	88	2.9%	95.7%		12	13.2%	13.6%	
** Fresenius Kabi USA LLC**	51	1.7%	92.7%		23	25.3%	45.1%	
** Akorn Inc**	48	1.6%	80.0%		8	8.8%	16.7%	
** All other companies**	1882	62.4%	29.6%					
**AWP unit price median (IQR)**	1.5 (0.4,11.7)			<0.001	2.7 (1.2,3.6)			0.106
**AWP package price median (IQR)**	106.4 (32.0,513.1)			<0.001	75.7 (30.2,118.6)			0.003
** Missing**	426	14.1%	88.6%					
**Companies per strength (median(IQR)**	1.0 (1.0, 3.0)				3.0 (2.0,4.0)			<0.001
**NDCs per company (median(IQR))**	5.0 (2.0,15.5)				22.0 (7.0,38.1)			0.023
**Company risk (median (IQR))**					7.0 (3.0, 11.0)			<0.001

### Shortages of schedule II injectable opioids in the US market

There were 1,001 active schedule II NDC injectable opioids in the US market. Of them, 86 (8.6%) were in shortage as of April 30, 2019 (Table 3). Generic drugs represented 96.0% (n = 961) of the active opioid NDCs and 91.9% (n = 79) of the active NDC shortages. Fentanyl citrate represented 48.0% (n = 480) of the schedule II injectable opioid NDCs but 22.1% (n = 19) of the NDC shortages; whereas, hydromorphone hydrochloride represented 23.6% (n = 236) of the schedule II injectable opioid NDCs but 36.0% (n = 31) of the NDC shortages. Three companies accounted for 81.4% of the shortages of schedule II injectable opioid NDCs in the market. Conversely, 770 (76.9%) of the schedule II injectable opioid NDCs were manufactured by companies that did not experience any instances of shortages during the study period.

**Table 3:** DEA schedule CII injectable opioid NDCs marketed and shortages, 2015–2019.

	NDCs	% CII injectable NDC Marketed	FDA Shortages	% FDA Shortages	% NDCs	p value
**NDCs**	1,001		86		8.6%	<0.001
**Strengths**	213		25		11.7%	
**Companies**	16		8		50.0%	
**Fixed-dose drug combinations**	371	37.1%				
**Generic drugs**	961	96.0%	79	91.9%	8.2%	0.078
**Unit dose**	1	0.1%				
**Active ingredient**						<0.001
** alfentanil hydrochloride**	11	1.1%				
** fentanyl**	5	0.5%				
** fentanyl citrate**	480	48.0%	19	22.1%	4.0%	
** hydromorphone hydrochloride**	236	23.6%	31	36.0%	13.1%	
** meperidine hydrochloride**	45	4.5%	1	1.2%	2.2%	
** methadone hydrochloride**	8	0.8%	2	2.3%	25.0%	
** morphine sulfate**	175	17.5%	27	31.4%	15.4%	
** remifentanil hydrochloride**	15	1.5%	6	7.0%	40.0%	
** sufentanil citrate**	26	2.6%				
**All companies with active NDCs in shortage**						<0.001
** Akorn, Inc**	21	2.1%	8	9.3%	38.1%	
** Fresenius Kabi USA, LLC**	51	5.1%	23	26.7%	45.1%	
** Hikma Pharmaceuticals USA Inc**	42	4.2%	12	14.0%	28.6%	
** Hospira, Inc**	104	10.4%	35	40.7%	33.7%	
** Mylan Pharmaceuticals, Inc**	7	0.7%	4	4.7%	57.1%	
** Teva Pharmaceuticals USA**	5	0.5%	3	3.5%	60.0%	
** All Other**	770	76.9%				
**AWP unit price median (IQR)**	0.3 (0.2, 0.8)		2.6 (1.0, 3.4)			<0.001
**AWP package price median (IQR)**	27.1 (16.6, 46.9)		63.0 (30.0, 106.9)			<0.001
** Missing**	89	8.9%				
**Companies per strength (median(IQR)**	1.0 (1.0, 1.0)		3.0 (2.0, 3.0)			<0.001
**NDCs per company (median(IQR))**	15.0 (3.5, 36.0)		18.0 (6.5, 37.2)			0.495
**Company risk (median (IQR))**	3.0 (0.0, 10.0)		7.5 (2.8, 11.5)			0.183

### Predictors of shortages of opioids

The risk of shortage was significantly greater for companies that experienced prior instances of NDC opioid shortages during the study period (OR 1.034; p < 0.01) (Table 4). For a unit increase in the number of prior instances of shortages of a given company, the likelihood of an NDC shortage increases by 3.4% for that company. In addition, the greater was the number of NDCs marketed by a company, the lower was the risk of shortages for that company (OR 0.99; p < 0.001). More specifically, for a unit increase in number of NDCs marketed by a company, the odds of an NDC shortage decreases by 1%. Compared to brands, the risk of shortage was greater for generic drugs, although not statistically significant. The number of companies marketing a given strength was also positively associated, but not statistically significant, with the risk of shortages. The H-L goodness of fit test p = 0.95 and McFadden pseudo R^2^ of 0.46 evidenced a good model fit. In addition, the accuracy of the model was evidenced with an area under curve (AUC) of 0.918 (0.8868–0.9491). For a ROC probability of 28.0% or greater, our model would accurately predict the likelihood of an NDC opioid to be listed in shortage by the FDA 87.0% of the time. The model would also accurately predict the likelihood of an NDC not to be in shortage. Lastly, the accuracy of the model was also confirmed using the validation set (S1 Table).

**Table 4:** Multivariable logistic regression model for predicting risk of shortage of opioid analgesics.

Covariates	Beta Estimate	Standard Error	Odds Ratio	Odds Ratio 95% Lower	Odds Ratio 95% Upper	P value
Intercept	-2.129	0.644	0.119	0.029	0.397	0.0009530
Generics	0.630	0.463	1.878	0.798	5.990	0.1730577
# Companies per strength	0.271	0.163	1.311	0.947	1.816	0.0966278
# NDCs per company	-0.008	0.002	0.992	0.945	0.995	0.0000798
Company risk	0.034	0.012	1.034	1.011	1.283	0.0057170

Hosmer-Lemeshow Statistic p-value:0.95

McFadden pseudo R^2^: 0.46

Area Under Curve (95% CI):0.918(0.8868–0.9491)

ROC probability cut-off: 0.28

## Discussion

This is the first study to comprehensively characterize the market of opioid analgesics and to identify predictors of shortages. We found that two thirds of the opioid NDCs listed by the FDA were discontinued from the market. Shortages disproportionally impacted schedule II and injectable opioids. A few companies and active ingredients accumulated a disproportionately large amount of shortages of opioid analgesics. Three companies accounted for more than 4 in 5 of the schedule II active injectable opioids. The market of schedule II injectable opioids is highly concentrated and filled with off patent products. Relatively low prices of opioid analgesics reduced manufacturers’ incentives to remain in the market. In addition, few companies have the capacity to produce sterile injectable products [15, 16]. Injectable products are costlier than oral medications, have less clinically interchangeable alternatives and potentially greater impact on patient care [17, 18].

We also found that the risk of shortage of an NDC significantly decreases with the total number of NDCs marketed by the company and increases with the incidence of previous instances of shortages of the manufacturing company. These findings suggest that the characteristics of the manufacturing company might be the missing piece to the complex puzzle of the drug shortages in the US market. Companies manufacturing a greater amount of opioid NDCs may have more resources and the know-how providing the company with a competitive advantage. On the other hand, manufacturing companies operate on a just-in-time basis with very limited capacity to react timely to any problems encountered during the manufacturing process including disruptions in the supply of raw materials, active pharmaceutical ingredients (API) and inactive excipients, limited number of API suppliers, staffing shortages, and sterility testing capacity. In addition, most opioid analgesics have been in the market for more than 30 years and manufacturing companies may have not kept up with maintenance of depreciated infrastructures and obsolete management systems. Limited manufacturing capacity and unforeseen production interruptions rapidly lead to drug shortages. The FDA recommendations to address drug shortages include companies developing redundancy, capability, and manufacturing capacity [19].

The FDA Drug Shortages Task Force identified lower-priced drugs and sterile injectables as being potentially more vulnerable to drug shortages [20]. Others suggested that a greater number of companies marketing a drug would increase competition and reduce the incidence of shortages. We found that generic opioids and the overall number of companies with active NDCs for a given strength were positively associated with the risk of shortage–although the association was not statistically significant. Companies marketing single source drugs may be able to set higher prices and therefore, have more incentives to prevent market disruptions. While, each drug company has an intrinsic risk of shortage, there might be an added competition effect where the risk of shortage among competitor companies increases. These findings shed light on the debate regarding the role of market competition in the incidence and impact of shortages of opioid analgesics.

Study findings also have several implications for clinical and pharmacy practice. Shortages of morphine, hydromorphine, fentanyl, remifentanil commonly used in postoperative pain management increase the risk of medication errors and perioperative complications [5]. Inpatient administration of therapeutic interchangeable drugs (fentanyl instead of morphine), enteral opioids, instead of intravenous opioids in patients who can tolerate oral medications, and dose rounding to reduce drug wastage may also be considered to prevent and mitigate shortages. Purchasing departments and pharmacy and therapeutic committees across the nation might consider the likelihood of a dug to be in short supply and the manufacturer specific risk as part of their purchasing and drug formulary deliberations. Procurement agreements may include contingency clauses, performance requirements, and failure-to-supply clauses, and low-price clauses and restrictions on limiting downstream price increases to maintain sustainability of the supply. Procurement and payment policies may be coupled with a requirement for manufacturers to establish risk management plans to respond efficiently and effectively to potential shortages.

In addition, drug shortages increase waste and healthcare cost to health plans, providers, and patients. Excess costs include opioid-related adverse events treatment cost, operation, and labor cost [4]. Pharmacists devote a significant amount of time to manage drug shortages, revise treatment protocols and adjust information technology systems and ensure proper clinicians’ education regarding alternative medications. Evidence of the financial impacts of drug shortages remains of paramount importance for purchasers and healthcare providers to make inform decisions and secure a reliable drug supply. Despite the incidence, persistence and impacts on healthcare delivery, most hospitals and other healthcare systems have not comprehensively quantified the cost of drug shortages [21].

This study has some limitations. Despite the high prevalence and significant impacts of shortages there is not a harmonized definition of drug shortages. The FDA defines a drug shortage as a situation in which the total supply of all clinically interchangeable versions of an FDA regulated drug is inadequate to meet the current or projected demand at the user level [22]. The FDA reports shortages of products medically necessary to treat or prevent a serious disease or medical condition for which there is no other appropriate and available substitute. The American Society of Health-System Pharmacists defines pharmaceutical shortages more broadly as a supply issue that affects how the pharmacy prepares or dispenses a drug product or how patient care is influenced when prescribers must use alternative products [23]. Furthermore, healthcare providers across the country may follow their own definitions of drug shortages when products are on back order, in allocation or not sufficient units remain in inventory to fulfill their target quantity. In addition, shortage dates vary by data source. We used the first date of an opioid analgesic was reported in shortage at the FDA website. An active ingredient may have several administration routes and strengths. Hence, our analysis at the NDC level, rather than the active ingredient, provides a finer characterization of the drug supply and therapeutic alternatives available to clinicians. Study variables with 20% or more missing observations were not included in the analysis (i.e., ANDAs and NDAs by active ingredient). Lastly, regression models may be subject to bias from unobserved market and drug-related characteristics including the Drug Enforcement Administration Annual Aggregate Production Quotas for schedule I and II opioids and manufacturers lack of compliance with Good Manufacturing Practices.

## Conclusions

Over eight thousand opioid NDCs were listed by the FDA for marketing in the US. Only one in three opioid analgesics approved by the FDA were active as of April 30, 2020. Four in five active opioids in the US market were schedule II opioids and generics. There were significant differences in the incidence of shortages by active ingredient and manufacturing company. Three companies accounted for four in five shortages of schedule II injectable opioid NDCs in the market. For a unit increase in the number of prior instances of shortages of a given company, the likelihood of an NDC shortage increases by 3.4% for that company. The greater the number of NDCs marketed by a company, the lower was the risk of shortages for that company. The overall number of companies marketing an opioid analgesic was not significantly associated with the risk of shortage.

## Supporting information

S1 Table DEA schedule CII injectable opioids in the market stratified by training and validation sets. (DOCX)
