# Supplementary material for: Predictors of shortages of opioid analgesics in the US: Are the characteristics of the drug company the missing puzzle piece?
Source: PLoS One. 2021 Mar 31;16(3):e0249274. doi: 10.1371/journal.pone.0249274 (PMC8011730; doi:10.1371/journal.pone.0249274)
Supplement: S1 Table — (DOCX) [file pone.0249274.s001.docx]

**S1 Table. DEA Schedule CII Injectable Opioids in the Market Stratified by Training and Validation Sets**

|  | **Training Dataset** | | | | | | | | | | | **Validation Dataset** | | | | | | | | | | |
| --- | --- | --- | --- | --- | --- | --- | --- | --- | --- | --- | --- | --- | --- | --- | --- | --- | --- | --- | --- | --- | --- | --- |
|  | **N** | **%** | | **FDA Shortages** | | **% of FDA Shortages** | | **% of NDC** | | **p value** | | **N** | **%** | | **FDA Shortages** | | **% of FDA Shortages** | | **% of NDC** | | **p value** | |
| **NDCs** | 702 |  | | 61 | |  | | 8.7% | |  | | 299 |  | | 25 | |  | | 8.4% | |  | |
| **Strengths** | 182 |  | | 24 | |  | | 13.2% | |  | | 114 |  | | 11 | |  | | 9.6% | |  | |
| **Companies** | 15 |  | | 7 | |  | | 46.7% | |  | | 12 |  | | 7 | |  | | 58.3% | |  | |
| **Shortages** | 61 | 8.7% | | 61 | | 100.0% | | 100.0% | | <0.001 | | 25 | 8.4% | | 25 | | 100.0% | | 100.0% | | <0.001 | |
| **Fixed-dose drug combination** | 258 | 36.8% | | 0 | | 0.0% | | 0.0% | | <0.001 | | 113 | 37.8% | | 0 | | 0.0% | | 0.0% | | <0.001 | |
| **Generic drugs** | 669 | 95.3% | | 54 | | 88.5% | | 8.1% | | 0.021 | | 292 | 97.7% | | 25 | | 100.0% | | 8.6% | | 0.906 | |
| **Unit dose** | 1 | 0.1% | | 0 | | 0.0% | | 0.0% | | 1 | | 299 | 100.0% | | 25 | | 100.0% | | 8.4% | | NA | |
| **Active ingredient** |  |  | |  | |  | |  | | <0.001 | |  |  | |  | |  | |  | | 0.094 | |
| **alfentanil hydrochloride** | 7 | 1.0% | | 0 | | 0.0% | | 0.0% | |  | | 4 | 1.3% | | 0 | | 0.0% | | 0.0% | |  | |
| **fentanyl** | 4 | 0.6% | | 0 | | 0.0% | | 0.0% | |  | | 1 | 0.3% | | 0 | | 0.0% | | 0.0% | |  | |
| **fentanyl citrate** | 327 | 46.6% | | 11 | | 18.0% | | 3.4% | |  | | 153 | 51.2% | | 8 | | 32.0% | | 5.2% | |  | |
| **hydromorphone hydrochloride** | 169 | 24.1% | | 23 | | 37.7% | | 13.6% | |  | | 67 | 22.4% | | 8 | | 32.0% | | 11.9% | |  | |
| **meperidine hydrochloride** | 35 | 5.0% | | 1 | | 1.6% | | 2.9% | |  | | 10 | 3.3% | | 0 | | 0.0% | | 0.0% | |  | |
| **methadone hydrochloride** | 6 | 0.9% | | 2 | | 3.3% | | 33.3% | |  | | 2 | 0.7% | | 0 | | 0.0% | | 0.0% | |  | |
| **morphine sulfate** | 124 | 17.7% | | 19 | | 31.1% | | 15.3% | |  | | 51 | 17.1% | | 8 | | 32.0% | | 15.7% | |  | |
| **remifentanil hydrochloride** | 13 | 1.9% | | 5 | | 8.2% | | 38.5% | |  | | 2 | 0.7% | | 1 | | 4.0% | | 50.0% | |  | |
| **sufentanil citrate** | 17 | 2.4% | | 0 | | 0.0% | | 0.0% | |  | | 9 | 3.0% | | 0 | | 0.0% | | 0.0% | |  | |
| **All companies with active NDCs in shortage** |  |  | |  | |  | |  | | <0.001 | |  |  | |  | |  | |  | | <0.001 | |
| **Akorn, Inc** | 11 | 1.6% | | 3 | | 4.9% | | 27.3% | |  | | 10 | 3.3% | | 5 | | 20.0% | | 50.0% | |  | |
| **Fresenius Kabi USA, LLC** | 41 | 5.8% | | 19 | | 31.1% | | 46.3% | |  | | 10 | 3.3% | | 4 | | 16.0% | | 40.0% | |  | |
| **Hikma Pharmaceuticals USA Inc** | 29 | 4.1% | | 8 | | 13.1% | | 27.6% | |  | | 13 | 4.3% | | 4 | | 16.0% | | 30.8% | |  | |
| **Hospira, Inc** | 72 | 10.3% | | 26 | | 42.6% | | 36.1% | |  | | 32 | 10.7% | | 9 | | 36.0% | | 28.1% | |  | |
| **Mylan Pharmaceuticals, Inc** | 7 | 1.0% | | 4 | | 6.6% | | 57.1% | |  | | 1 | 0.3% | | 1 | | 4.0% | | 100.0% | |  | |
| **Teva Pharmaceuticals USA** | 2 | 0.3% | | 1 | | 1.6% | | 50.0% | |  | | 3 | 1.0% | | 2 | | 8.0% | | 66.7% | |  | |
| **All Other** | 540 | 76.9% | | 0 | | 0.0% | | 0.0% | |  | | 230 | 76.9% | | 0 | | 0.0% | | 0.0% | |  | |
| **AWP unit price** |  | |  | |  | |  | |  | |  |  | |  | |  | |  | |  | |  |
| **Median (IQR)** | 0.3 (0.2, 0.8) | |  | | 2.9 (1.2, 4.1) | |  | |  | | <0.001 | 0.4 (0.2, 0.8) | |  | | 2.5 (0.8, 3.0) | |  | |  | | <0.001 |
| **Missing** | 67 | | 9.5% | | 0 | | 0.0% | | 0.0% | | 0.015 | 22 | | 7.4% | | 0 | | 0.0% | | 0.0% | | 0.284 |
| **AWP package price** |  | |  | |  | |  | |  | |  |  | |  | |  | |  | |  | |  |
| **Median (IQR)** | 26.7 (15.9, 46.2) | |  | | 75.7 (30.0, 108.0) | |  | |  | | <0.001 | 29.2 (17.4, 48.9) | |  | | 62.0 (29.9, 91.5) | |  | |  | | <0.001 |
| **Missing** | 67 | | 9.5% | | 0 | | 0.0% | | 0.0% | | 0.015 | 22 | | 7.4% | | 0 | | 0.0% | | 0.0% | | 0.284 |
| **Companies per strength (median(IQR))** | 1.0 (1.0, 1.0) | |  | | 2.5 (2.0, 3.0) | |  | |  | | <0.001 | 1.0 (1.0, 2.0) | |  | | 3.0 (2.0, 3.5) | |  | |  | | <0.001 |
| **NDCs per company(median(IQR))** | 15.0 (5.0, 36.0) | |  | | 21.0 (11.0, 38.5) | |  | |  | | 0.349 | 19.0 (5.8, 39.8) | |  | | 21.0 (10.0, 38.5) | |  | |  | | 0.928 |
| **Company risk (median(IQR))** | 4.0 (0.0,11.0) | |  | | 8.0 (5.0, 12.0) | |  | |  | | 0.128 | 7.5 (7.5, 11.8) | |  | | 8.0 (4.5, 12.0) | |  | |  | | 0.433 |
